# Assessing stroke survivors’ knowledge: a scoping review of tools and influencing factors

**DOI:** 10.1186/s41043-025-01046-3

**Published:** 2025-09-26

**Authors:** Allam Harfoush, Kausik Chatterjee, Hanady Hamdallah

**Affiliations:** 1 The Faculty of Health, Medicine, and Society, University of Chester, Chester, CH1 4BJ UK; 2 North Bristol NHS Foundation Trust, Southmead Road, Bristol, BS10 5NB UK; 3 Aintree University Hospital, Lower Ln, L9 7AL Fazakerley, Liverpool, UK; 4 UCL Primary Care & Population Health, UCL, Gower St, London, WC1E 6BT UK

**Keywords:** Cerebrovascular disorders, Health knowledge, Attitudes, Practice, Patient education

## Abstract

**Background:**

Stroke survivors’ knowledge of their condition is essential for self-management and adherence to secondary prevention strategies. However, current methods for assessing stroke knowledge are not consistent. This scoping review aimed to review existing assessment tools, evaluate their characteristics, and identify factors associated with stroke knowledge to inform the development of more effective, patient-centred educational strategies.

**Review design and methods:**

A scoping review was conducted to evaluate existing methods used to assess stroke knowledge and the factors influencing patients’ knowledge among stroke survivors. Systematic searches of PubMed, Cochrane, and CINAHL were performed from inception to June 2025. Studies assessing stroke survivors’ knowledge were included. Data were extracted on tool characteristics, assessed themes, administration methods, readability, validation, and knowledge-associated factors. Findings were qualitatively synthesised.

**Results:**

Thirty-nine studies were included. Most studies assessed mixed cohorts of ischaemic, haemorrhagic, and TIAs. Stroke symptoms and risk factors were the most frequently assessed themes, while rehabilitation, medications, and lifestyle behaviours were less explored. Tools were primarily self-administered questionnaires, typically completed in under 15 min, but often lacked standardised cut-off values and demonstrated limited reporting of development processes. Factors positively associated with knowledge included higher education, younger age, and healthier lifestyles.

**Conclusion:**

Existing assessments of stroke survivors’ knowledge have considerable variability, limited validation, and inconsistent alignment with survivors’ information needs. Developing standardised, validated, and patient-centred assessment tools that are tailored to stroke type and accessible across literacy levels is essential for advancing stroke education and supporting long-term recovery. These findings can inform policymakers in tailoring education efforts and designing interventions that directly address knowledge gaps across diverse stroke survivor populations. Future research should prioritise longitudinal evaluation of knowledge and its impact on clinical outcomes.

**Supplementary Information:**

The online version contains supplementary material available at 10.1186/s41043-025-01046-3.

## Background

Stroke is one of the leading causes of death and long-term disability worldwide. According to the World Stroke Organisation, over 12 million people experience a stroke each year [1]. Beyond its physical and cognitive effects, stroke places a significant economic and social costs [2]. This burden is particularly evident in the UK, where the annual cost exceeds £26 billion [3]. Furthermore, nearly 40% of strokes in the UK affect individuals of working age, contributing to reduced workforce participation and increased demand on social support [4].

Although medical advancements have improved acute stroke care, effective recovery and secondary prevention require strategies that go beyond immediate clinical interventions. Optimal post-stroke management depends on survivors’ active involvement in their treatment plans [5]. Patients who are well-informed are more likely to engage in their care, make informed decisions, and integrate preventive measures into daily routines [6–8].

However, despite its importance, stroke survivors’ knowledge remains an often-overlooked component of long-term care. Knowledge gaps persist due to inconsistent information delivery, limited time for patient education in clinical settings, and variability in the quality of educational resources [9]. This suggests the need to review existing assessment tools to inform the development of high-quality tools to be aligned with the emerging field of precision medicine [10]. While previous reviews have summarised the methods used to assess stroke knowledge, factors associated with knowledge have not been comprehensively reviewed. Therefore, this scoping review was conducted to address the following questions: What tools are currently used to assess stroke survivors’ knowledge, and what are their characteristics and what individual and contextual factors are associated with variations in stroke knowledge among survivors. By answering these questions, this review aims to inform the design of standardised, validated, and patient-centred assessment tools and support the development of targeted education strategies for stroke survivors.

## Literature search

### Search strategy

A literature search was undertaken across three databases, PubMed, Cochrane Library, and CINAHL, from inception till June 2025. The search strategy included the following keywords (stroke[Title/Abstract] OR cerebrovascular disease[Title/Abstract] OR transient ischaemic attack[Title/Abstract] OR TIA[Title/Abstract]) AND (education[Title/Abstract] awareness[Title/Abstract] OR knowledge[Title/Abstract] OR understanding[Title/Abstract]) AND (questionnaire[Title/Abstract] OR scale[Title/Abstract] OR survey[Title/Abstract]). The search strategy was piloted first by trailing several combinations of keywords. No language restrictions or additional database filters were applied at the initial search stage. Manual screening of the reference lists of the included studies was also used to identify any further relevant studies (other methods in the PRISMA flow chart).

### Selection criteria

Before the search, a set of inclusion and exclusion criteria was established. The inclusion criteria were studies evaluating stroke survivors’ disease knowledge at any stage of their condition, involving participants aged 18 or older. While studies exclusively assessing stroke knowledge in the general public without inclusion of stroke survivors, studies that included interventions, studies that focused on a certain non-stroke focused aspect of knowledge (e.g. obstructive sleep apnoea, depression) were excluded. Grey literature, such as unpublished theses, conference abstracts, and institutional or government reports, was excluded to maintain a focus on studies that had undergone formal peer review, to ensure a minimum level of methodological transparency and reporting quality. This decision was made because such sources often lack sufficient detail on study design, sample characteristics, and statistical analysis, making it difficult to assess the validity of findings or include them meaningfully in synthesis, which could compromise the consistency and reliability of the review’s findings. However, we acknowledge that excluding grey literature may have led to disregarding of potentially relevant outcomes and we do acknowledge the potential for publication bias in this review. Similar exclusion criterion has been applied in other literature reviews on stroke-related knowledge and education [55].

### Data extraction

The studies were screened based on their titles and abstracts to identify eligible publications. If the title and abstract were inconclusive, a thorough full-text review was conducted. Additionally, duplicates were manually removed by cross-referencing the title, publication date, author names, and abstracts. Selection procedures adhered to the Preferred Reporting Items for Systematic Reviews and Meta-Analyses (PRISMA) guidelines.

### Themes of the scoping review

Data were extracted using a structured Excel sheet, collecting study- and participant-related characteristics. At the study level, extracted data included the country of origin, sample size, and included population for each study. The assessment tools used to measure stroke knowledge were also recorded, including the tool’s name, year of development, and the method by which it was created. Additionally, the themes covered by each tool, administration method, and, where available, the number of questions, question type, and Flesch readability score were also documented. Moreover, data on whether the tool had any form of optimisation to assess reliability and validity were included where available. In addition, participant-related variables that associated with stroke knowledge were extracted, including demographic, medical, and lifestyle factors.

### The rationale behind using narrative synthesis

Although meta-analysis would be a valuable method for synthesising quantitative findings across studies, it was not appropriate in this review due to substantial heterogeneity across included studies. Specifically, the variability in outcome measures, statistical reporting, and methodological approaches. Firstly, there was considerable variation in how stroke awareness and symptom recognition were reported. For example, Wongwiangjunt et al., 2015 [29] and Mosley et al., 2013 [33] reported adjusted odds ratios (ORs) with 95% confidence intervals for factors associated with stroke awareness, enabling direct statistical comparison. In contrast, Slark et al., 2012 [34] and Sloma et al., 2010 [36] presented only descriptive data in the form of percentages, such as the proportion of participants recognising FAST symptoms or identifying stroke risk factors, without statistics or variance estimates. This lack of effect size reporting meant that essential statistical inputs for a meta-analysis were unavailable. Secondly, the inconsistency in statistical reporting further limited synthesis. While Wongwiangjunt et al., 2015 [29] and Mosley et al., 2013 [33] used multivariable logistic regression, Widjaja et al., 2021 [15] reported chi-square test results and Spearman’s correlation coefficients, without ORs or standard errors. Soto-Cámara et al., 2020 [16] provided raw frequencies and p-values for symptom recognition, but insufficient data to reliably calculate or standardise effect sizes across studies. Furthermore, numerical reporting formats varied widely, with some studies presenting mean ± SD, others reporting median with interquartile range, and some not reporting obvious numerical outcomes. While statistical methods exist to approximate means and SDs from medians and IQRs [11], these rely on assumptions of normality that may not hold for knowledge data, which is often skewed [12]. Additionally, pooling results across incompatible measurement scales would risk producing misleading outcomes. Due to these methodological and statistical inconsistencies, a narrative synthesis was chosen as the most appropriate approach to integrate and interpret the available evidence.

## Overview of reviewed studies and factors influencing stroke knowledge

**Fig. 1:**
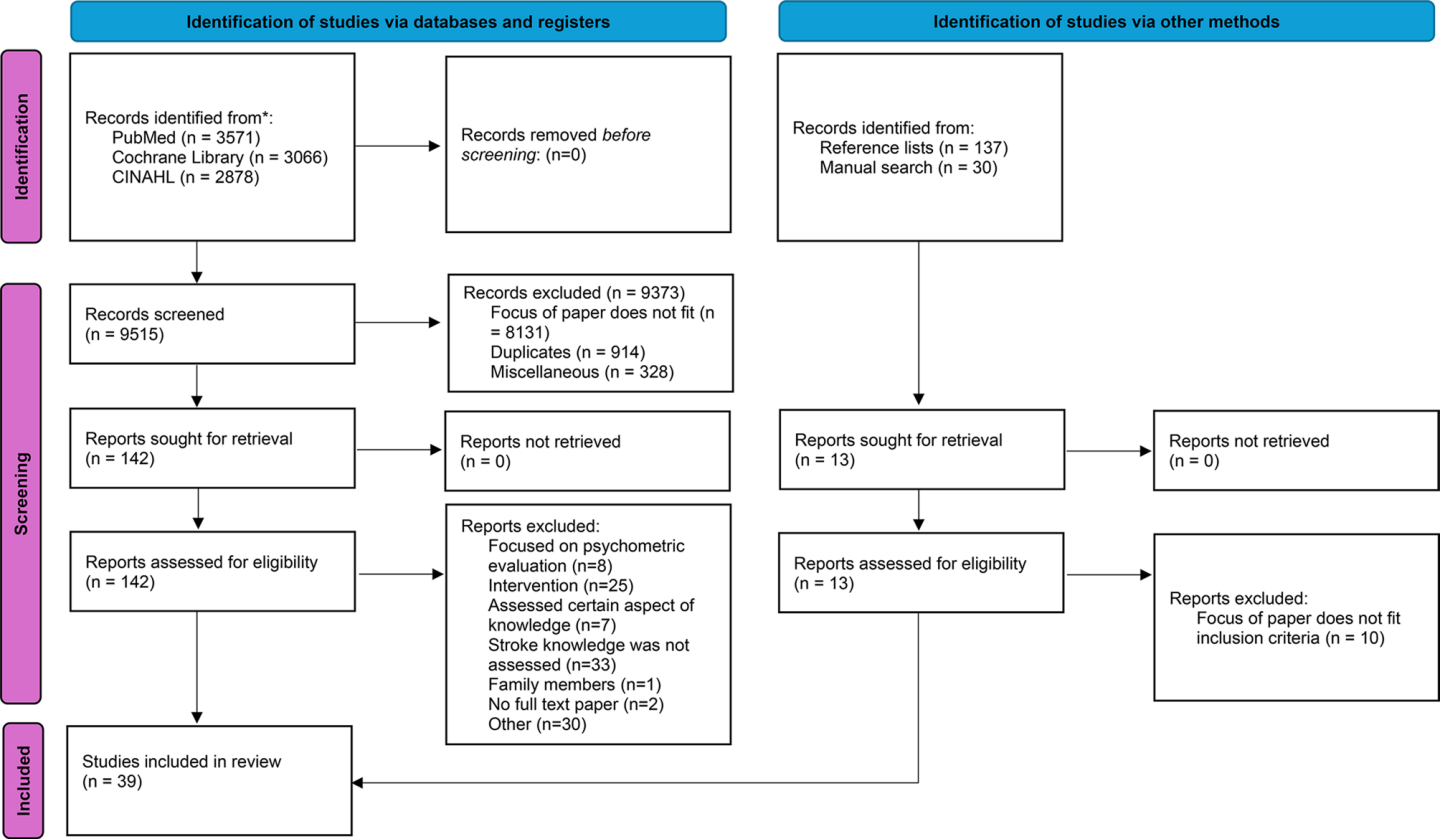
The PRISMA flowchart of this scoping review

Thirty-nine studies published between 1997 and 2024 (Fig. 1, PRISMA flowchart) were included. Table 1 summarises the study characteristics and the tools used to assess stroke knowledge.

### Characteristics of the studies

#### The targeted population

The studies included a diverse population, mostly patients with haemorrhagic and ischaemic strokes, as well as transient ischaemic attacks (TIAs). However, three studies focused exclusively on patients with ischaemic strokes [22, 25, 30], one study on individuals with only TIAs [38], and another on patients with recurrent stroke [15]. This diversity could lead to overgeneralisation, as each group has different stroke aetiology, risk factors, and management plans [52]. For example, ischaemic strokes are often associated with atherosclerosis and cardioembolic events, while haemorrhagic strokes are more commonly linked to hypertension and vascular malformations [53], suggesting that a one-size-fits-all approach may overlook important differences in patients’ knowledge across stroke types.

Additionally, some studies recruited participants within the first 48 h of admission. Cognitive deficits such as memory loss, confusion, and reduced attention are common in the acute phase of stroke and can impact comprehension, which can underestimate survivors’ true knowledge [54]. Additionally, most reviewed studies excluded survivors with aphasia and communication disorders. The observed heterogeneity highlights the need for future research to adopt more homogeneous samples, focusing on specific stroke types to improve the interpretability of findings.

**Table 1:**
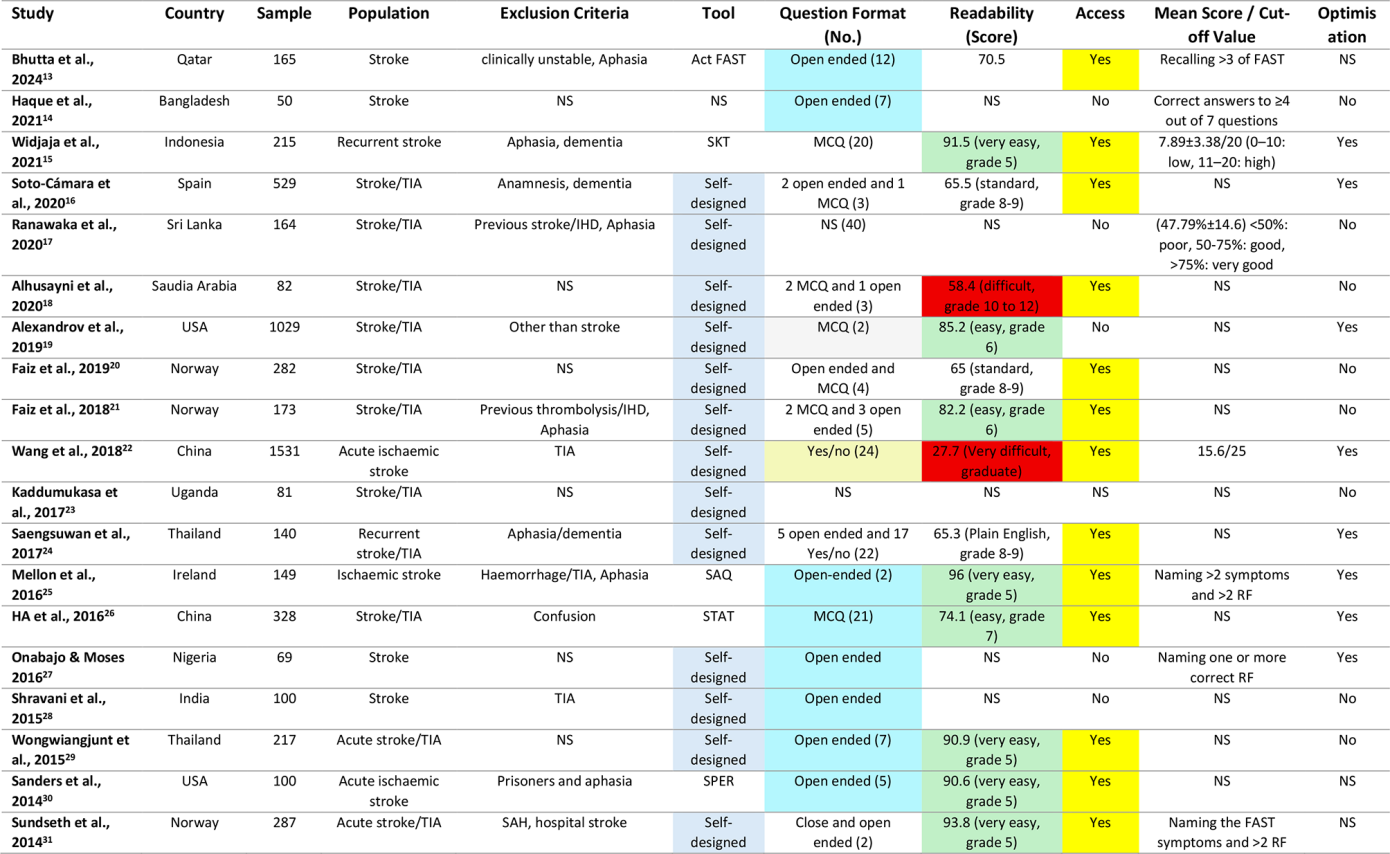

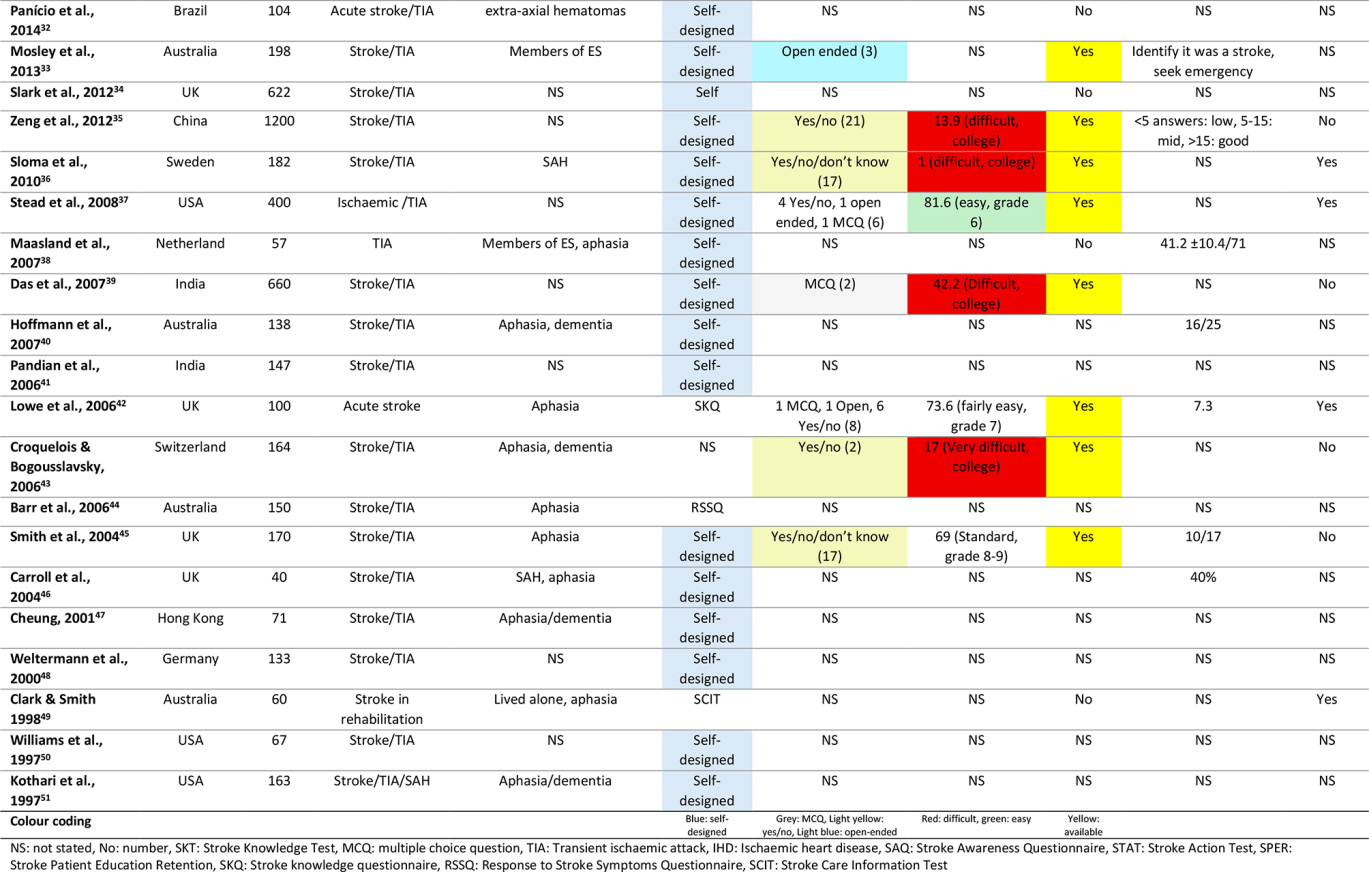
The outcomes of the reviewed studies

#### The explored themes

**Fig. 2:**
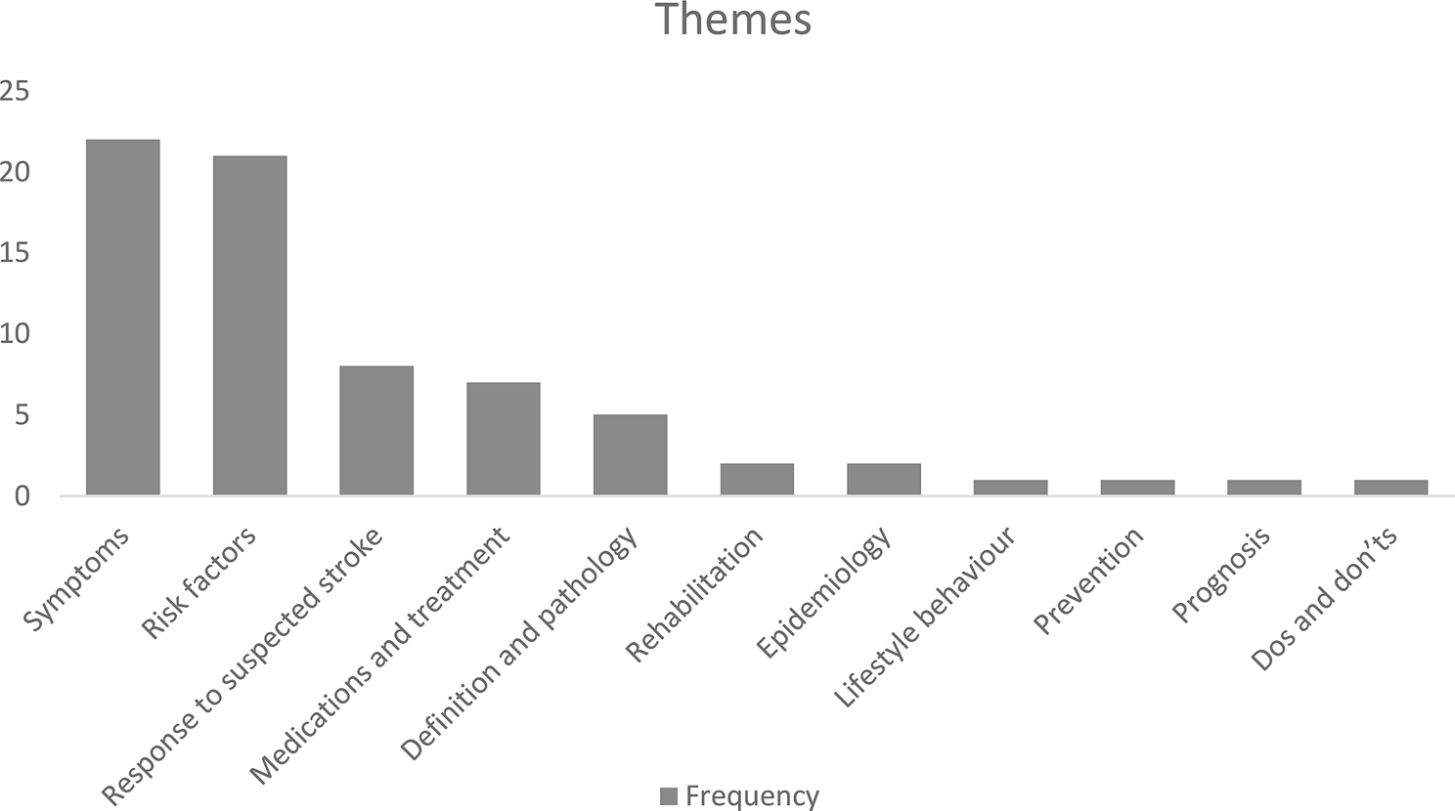
The frequency of the explored themes in our review

While stroke symptoms and risk factors were the most commonly assessed themes (Fig. 2, which shows that certain themes are less explored in the literature), other equally important areas, such as rehabilitation, medications, and lifestyle behaviours, received less attention. This narrow focus may limit the clinical effectiveness of information, as survivors often face challenges navigating post-discharge care without adequate support and potentially leading them to social isolation [8, 55, 56]. A broader exploration of such themes could better identify knowledge gaps and highlight areas where survivors need support, positively impacting their recovery and long-term health.

#### Method of administration

Most studies used self-administered questionnaires, reflecting their convenience and cost-effectiveness. In contrast, interviews, though helpful for clarifying responses, were less common due to higher costs and logistical complexity. In the literature, the method of administration (self-administered vs. interviews) generally did not significantly affect questionnaire scores [57]. However, self-administered tools tend to be more reliable for sensitive topics [58], as respondents may feel more comfortable and honest without an interviewer present, reducing social desirability bias and improving response accuracy.

#### Questionnaire format & administration time

While completion time data was not available for most studies, those reported it had an average time of less than 15 min. This suggests a design focused on conciseness and user-friendliness, which can increase participant engagement and minimise respondent fatigue, thus supporting higher-quality responses [59]. This is especially important in clinical and research settings where time and participant adherence are essential. However, such brevity may limit the depth of information collected, requiring a balance between efficiency and comprehensiveness.

The number of questions across studies varied widely, from 2 to 40, and included formats such as yes-or-no, multiple-choice (MCQs), and open-ended questions. This reflects variable research aims and contexts, which affects the scope and depth of each questionnaire, as too many questions or overly complex formats may lead to respondent fatigue and lower completion rates [59]. Each questionnaire format has advantages and disadvantages. Yes-or-no formats are straightforward and quick, ideal for gathering basic information or assessing binary outcomes [60]. Multiple-choice questions allow a broader range of responses, enabling a more detailed assessment of understanding and reducing guesswork compared to yes-or-no formats [61]. Open-ended questions allow in-depth knowledge assessment but can be more challenging to analyse quantitatively [62].

The number and type of questions should align with the study’s objectives. Researchers might favour yes-or-no questions for basic knowledge, while deeper insights may require multiple-choice or open-ended formats [63]. Consideration of the target population and administration context is also essential; for example, shorter, simpler questionnaires may be more suitable for individuals with cognitive or reading difficulties.

#### Tool reporting, readability, and validation

Most questionnaires were not published in the original articles or their appendices, creating challenges for researchers looking to build on these studies’ findings. While some studies gave descriptions or question examples in the methodology sections, this does not replace accessing the full questionnaires. Sharing full questionnaires ensures consistent data collection, facilitates cross-study validation, and enables future research to build on robust foundations. All available extracted questionnaires are presented in Appendix A.

Most included questionnaires scored ≥ 60 on the Flesch scale (grade 8–9), indicating general readability [64]. However, some exceeded grade 9, posing potential challenges for stroke survivors, who often read at grade 7–8 levels [65]. Readability is essential, as complex language causes misunderstandings, incomplete responses, and less reliable data [66], especially that stroke survivors may have visual and reading difficulties [67].

Only seven questionnaires from the reviewed studies specified cut-off values for knowledge scores. These values categorise varying knowledge levels and inform educational interventions. However, the methods used to determine these cut-off values were not reported, which is essential to accurately interpret knowledge assessments and identify individuals who require additional educational input [68]. This should include statistical justifications, evidence from prior research, or expert opinions.

Nearly half of the tools used did not report the optimisation process. This process confirms that the questionnaire is clear, reliable, and valid before full administration [69]. Addressing these problems beforehand reduces the risk of data collection errors. Additionally, it allows researchers to test logistical aspects of the study, including completion time, ease of administration, and the overall data collection process [70].

### Factors affecting stroke survivors’ knowledge

**Fig. 3:**
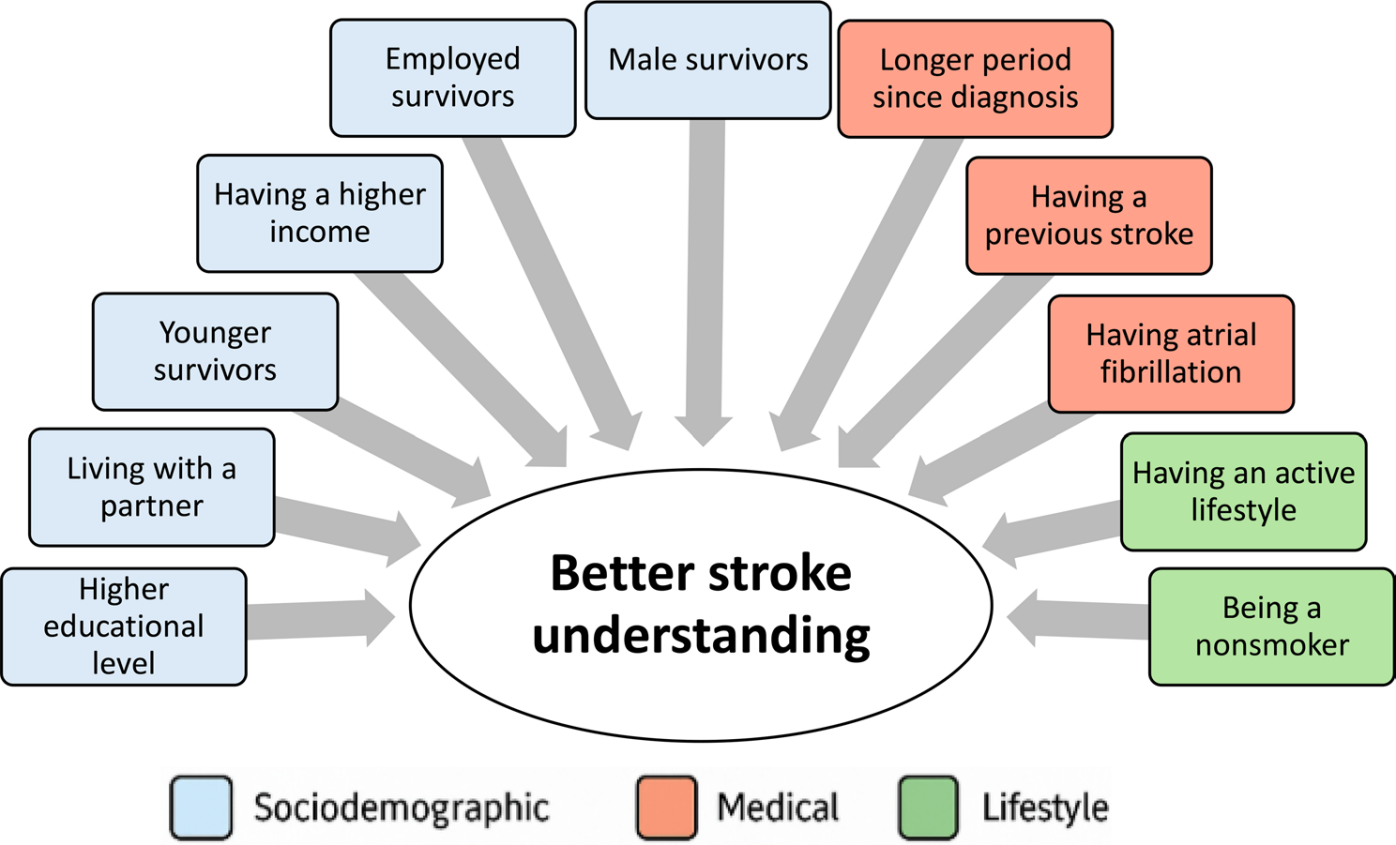
Factors influencing knowledge levels

We identified several factors influencing survivors’ knowledge, categorised into three groups: sociodemographic, medical, and lifestyle-related factors. As illustrated in Fig. 3, stroke knowledge is shaped by a combination of elements such as education, social support, health behaviours, and medical history. However, generalising these results is challenging since each assessment tool evaluated different aspects of stroke knowledge. Nonetheless, our findings may reflect a general trend regarding these factors, with a detailed presentation provided in Table 2. During data extraction, we intended to conduct a meta-analysis of factors influencing knowledge using the data in Table 2, but methodological and statistical heterogeneity prevented this. Many studies lacked sufficient detail, with inconsistent reporting and missing statistics such as standard deviations and confidence intervals. Consequently, we performed a qualitative synthesis, identifying three categories of influencing factors: sociodemographic, medical, and lifestyle-related (Fig. 3). These factors emerged as significant across several studies, despite variations in design, sample size, and methodology, suggesting a potential trend.

#### Sociodemographic-related (Blue)

There is a positive association between higher education levels and increased stroke knowledge, as educated individuals are more likely to understand and use health information effectively, adopt healthier lifestyles, and access health-related resources [71]. Additionally, patients living with a partner tend to have a deeper knowledge of stroke due to the support provided by their partners [72]. This support can lead to better communication, emotional support, and assistance in managing health-related issues. Younger survivors often engage more actively in managing their health by seeking information, while older patients may rely more on healthcare providers for decision-making [73]. Furthermore, individuals with higher incomes typically have better access to education and healthcare services, improving their stroke knowledge [74]. Employment is also linked to reduced social isolation, contributing to better disease knowledge by providing social interaction and access to information [75]. Lastly, male survivors can have greater access to and use of health information [76], which can be influenced by various social, cultural, and economic factors.

Additional underexplored area identified in this review is the role of cultural and regional differences in shaping stroke survivors’ knowledge.

#### Medical-related factors (Red)

Individuals with a history of stroke might have better knowledge due to their prior experiences with the warning signs and necessary lifestyle adjustments. Experiencing a stroke firsthand can motivate survivors to learn more about the condition to prevent recurrence. Additionally, a longer period since diagnosis could lead to better knowledge, as survivors gradually adapt to managing the condition and typically become more familiar with essential lifestyle changes, treatment, and risk factors over time [77]. Those managing atrial fibrillation also tend to engage in proactive information-seeking behaviours, due to the complexities of managing it, and since it increases stroke risk, people are often motivated to seek detailed information on both conditions [78].

#### Lifestyle-related factors (Green)

Smokers tend to show lower levels of stroke knowledge, possibly due to reported limitations in literacy when compared to non-smokers [79]. Smoking has been associated with negative health outcomes and cognitive decline, which may impair the ability to retain health information. Similarly, physical activity often correlates with proactive health-seeking behaviours, leading to higher levels of health knowledge [80]. Those who engage in regular physical activity may manage their health more actively.

The development of a formal conceptual framework to illustrate the interactions among these factors is planned as a subsequent step, building on the findings presented here.

**Table 2:**
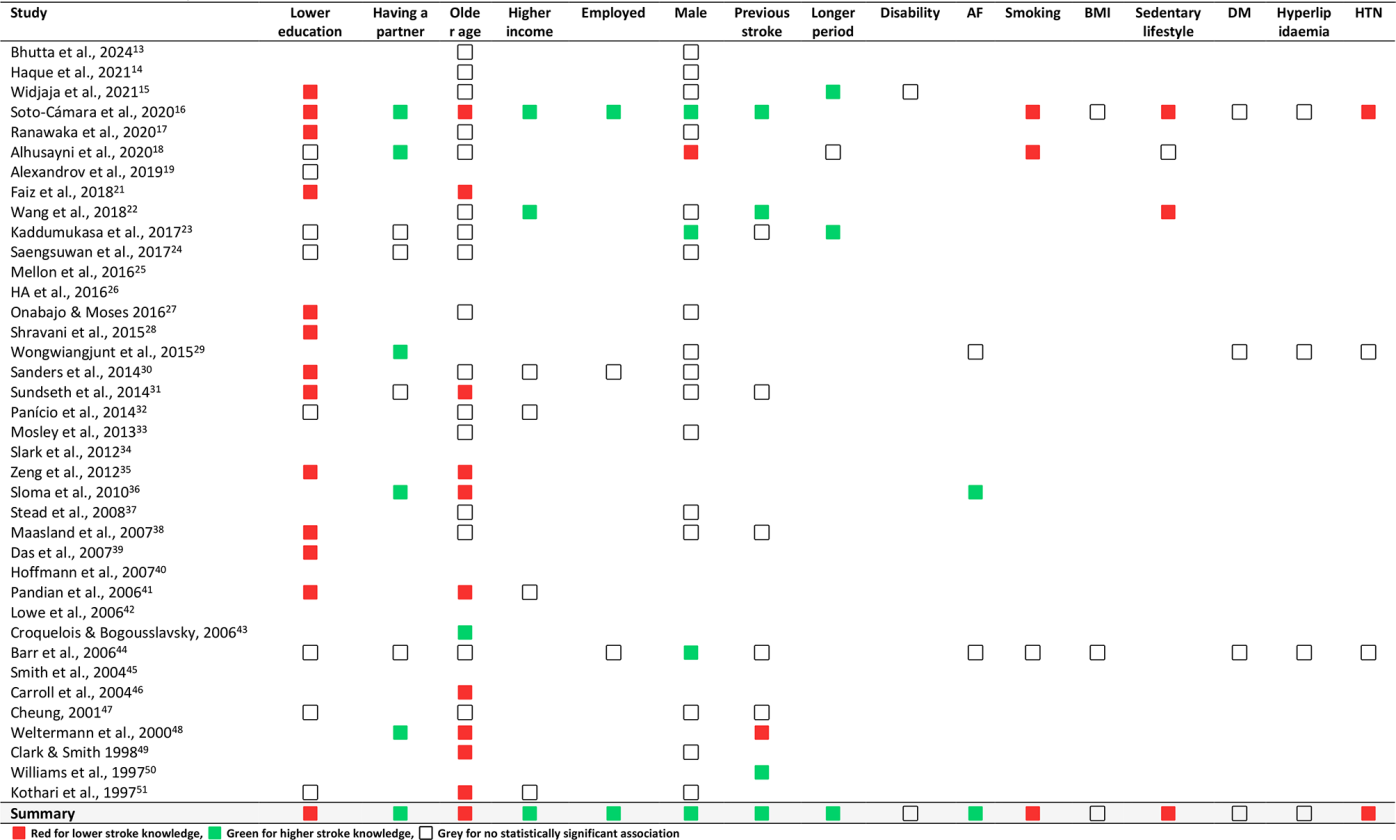
The knowledge outcomes in the included studies

## Identified gaps and proposed actions

Based on this scoping review, several gaps in existing assessments of stroke survivors’ knowledge were identified

First, the concept of “stroke survivor knowledge” remains inconsistently defined across studies, making it difficult to compare findings [81]. We propose conceptualising this concept prior to designing the methodology to evaluate stroke survivors’ knowledge. In our future work, we propose a definition that involves two core elements: self-management, reflecting survivors’ ability to independently manage their condition [82, 83], and relevant information, aligned with survivors individualised information needs [84, 85]. Importantly, many existing assessments did not adequately reflect on survivors’ real-world priorities and focused narrowly on symptoms and risk factors, neglecting equally important domains such as medications, rehabilitation, and lifestyle behaviours.

Second, current assessment tools often take a one-size-fits-all approach, overlooking the distinct needs of survivors with different stroke types. Additionally, few studies clearly report how knowledge cut-off values were determined, limiting the ability to identify those who would benefit from targeted interventions. We propose tailoring questionnaires for different stroke types and applying transparent, standardised statistical methods to define cut-off values (e.g. using the ROC curve method).

Finally, the reporting and accessibility of tools remain limited, with many studies failing to publish full questionnaires, detail their optimisation processes, or assess readability. Furthermore, important sociodemographic and behavioural factors, such as education, employment, smoking, and physical activity, were not consistently included, despite their potential influence on health knowledge.

Based on the limitations identified in this review, a future gold-standard stroke knowledge assessment tool should differ from existing instruments in several area. First, it should have clearly defined and theory-informed conceptual framework of stroke knowledge. Second, it should include a broader set of domains, like stroke symptoms, risk factors, rehabilitation, medications, and lifestyle behaviours, reflecting the real-world priorities of survivors. Third, the tool should be tailored to stroke type (e.g., ischaemic vs. haemorrhagic) and designed with accessible readability thresholds (ideally at a Grade 7 level). Fourth, psychometric validation (e.g., construct validity, internal consistency) and transparent statistical techniques (e.g., ROC analysis to determine cut-off scores) should be added into the development process. Finally, it should systematically include sociodemographic and behavioural data to support risk stratification and targeted education strategies. These considerations are essential for developing a valid, patient-centred, and clinically useful tool that addresses current gaps and supports tailored post-stroke education.

## Limitations, future direction, and clinical practice implications

This review has several limitations. First, the exclusion of grey literature may have led to missing valuable insights from non-peer-reviewed sources, such as clinical reports or government publications. Second, only English-language studies were included, which may have introduced language bias and limited the representation of stroke knowledge assessment practices from non-English-speaking regions. Third, several included studies had relatively small sample sizes, which may limit the statistical power and generalisability of their findings. Additionally, many studies lacked detail on questionnaire development or optimisation, affecting our ability to fully evaluate tool validity or reproduce their methodology. Finally, although a meta-analysis could have provided quantitative estimates of associations between influencing factors and stroke knowledge, the substantial heterogeneity in study designs, measurement tools, and reporting formats precluded direct comparisons and limited the generalisability of our findings.

Future research should prioritise the development of a validated assessment tool with clearly defined domains and appropriate psychometric properties. Studies should also explore and evaluate tailored educational interventions for stroke survivors that implement emerging technologies, such as artificial intelligence, to personalise content based on individual knowledge gaps, cognitive and literacy levels, and learning preferences. Longitudinal designs could help assessing the sustained impact of such interventions on knowledge retention, adherence to secondary prevention strategies, functional recovery, and recurrence rates over time. In addition, future work should address the current gaps in accessibility and inclusivity by making full assessment tools available through appendices or supplementary materials to facilitate replication and refinement, and by including diverse survivor populations, particularly those from underrepresented socioeconomic and geographic groups. Finally, and to enable direct quantitative syntheses in the future, researchers should report statistical outcomes fully and consistently.

The findings of this review have direct implications for clinical practice. The identified gaps in knowledge domains, such as rehabilitation, highlight areas where targeted educational interventions can be developed to address survivors’ information needs. Moreover, understanding how sociodemographic and medical factors influence knowledge might allow clinicians to tailor education based on patient characteristics, thereby improving engagement and supporting personalised recovery pathways.

## Conclusion

This scoping review mapped and evaluated existing tools for assessing stroke survivors’ knowledge, suggesting substantial variation in reliability, validity, and content domains. While the Stroke Knowledge Test demonstrated the strongest psychometric profile, no universally reliable and standardised instrument currently exists. This represents the need for a rigorously validated, adaptable tool grounded in precision medicine principles. The findings of this review can guide policymakers in selecting or developing more effective, inclusive, and evidence-based tools to improve patient education. Future research is needed to develop and validate a standardised, adaptable knowledge assessment tool and to examine how improved knowledge retention may influence health outcomes.

## Supplementary Information

Below is the link to the electronic supplementary material.

Supplementary Material 1

## Data Availability

No datasets were generated or analysed during the current study.
