# Supplementary material for: Assessing stroke survivors’ knowledge: a scoping review of tools and influencing factors
Source: J Health Popul Nutr. 2025 Sep 26;44:327. doi: 10.1186/s41043-025-01046-3 (PMC12465599; doi:10.1186/s41043-025-01046-3)
Supplement: Supplementary file 1 — Supplementary Material 1 [file 41043_2025_1046_MOESM1_ESM.docx]

| **Appendix A: the reported questionnaire** | | | | | |
| --- | --- | --- | --- | --- | --- |
| **Widjaja et al., 2020** | | | **1. The most common type of stroke occurs when**  (a) The blood supply to the brain is blocked  (b) You are having a heart attack  (c) There is bleeding in the brain  (d) You've had too much sun  (e) I don't know  **2. Which of the following will double your risk of stroke?**  (a) If you are asthmatic  (b) If you are diabetic  (c) If you exercise too much  (d) All of the above  (e) I don't know  **3. A type of irregular heartbeat known as Atrial Fibrillation (AF)**  (a) Decreases the risk of stroke  (b) Doubles the risk of stroke  (c) Increases the risk of stroke by more than 5 times  (d) Is not a risk factor of stroke  (e) I don't know  **4. Which age group is more at risk of stroke?**  (a) 20-30  (b) 31-50  (c) 51-60  (d)61+  (e) I don't know  **5. The warning signs of Transient Ischaemic Attack (TIA) disappear**  (a) Within 24 hours  (b) Within 48 hours  (c) After several days  (d) After several years  (e) I don't know  **6. Which of the following is a warning sign of stroke?**  (a) Sudden blurred vision  (b) Paralysis on one side of the body  (c) Severe headache  (d) All of the above  (e) I don't know  **7. For someone who has had a stroke, the main purpose of rehabilitation is to**  (a) Make sure they don’t take drugs  (b) Keep them in hospital as long as possible  (c) Improve their level of daily functioning  (d) Keep their mind off it  (e) I don’t know  **8. Taking aspirin assists in preventing stroke by**  (a) Stopping the formation of blood clots  (b) Getting rid of a headache  (c) Settling your stomach  (d) Relieving stress  (e) I don’t know  **9. You are at greater risk of stroke if**  (a) You are obese  (b) You exercise regularly  (c) You give up smoking  (d) All of the above  (e) I don't know  **10.Once you have suffered a Transient Ischemic Attack (TIA)**  (a) You are less likely to have a major stroke  (b) You are more likely to have a major stroke  (c) You are less likely to have a heart attack  (d) You are more likely to have a heart attack  (e) I don't know | **11.Surgery can sometimes help to prevent another stroke by**  (a) Giving a transfusion  (b) Cutting off the supply of blood to the brain  (c) Unblocking the arteries in the neck  (d) Removing the arteries  (e) I don't know  **12.What method of treatment is available for people who have had a stroke?**  (a) Medication  (b) Rehabilitation  (c) An operation  (d) All of the above  (e) I don't know  **13.The most important known risk factor for stroke is**  (a) Genetic  (b) Heart attack  (c) High blood pressure  (d) Old age  (e) I don't know  **14.Approximately how many Australians are affected by stroke every year?**  (a) 500  (b) 1 000  (c) 10 000  (d) 50 000  (e) I don't know  **15. If you drink alcohol excessively you are**  (a) Less likely to have a stroke  (b) Twice as likely to suffer stroke  (c) Three times as likely to suffer stroke  (d) Four times as likely to suffer stroke  (e) I don't know  **16.Which of the following is an example of a physical disability caused by stroke**  (a) The right arm is paralysed  (b) There are problems with memory  (c) Unable to speak properly  (d) Having trouble doing things in the correct order  (a) I don't know  **15.To reduce the risk of stroke you need to**  (a) Eat well and exercise regularly  (b) Ensure your blood pressure is not too high  (c) Monitor your cholesterol levels  (d) All of the above  (e) I don't know  **18.Smoking 20 cigarettes per day increases the risk of stroke by**  (a) 2 times  (b) 4 times  (c) 6 times  (d) 8 times  (e) I don't know  **19.If someone has a stroke, when should you ring for an ambulance?**  (a) Only ring if the symptoms stay after 24 hours  (b) Always ring for an ambulance straight away  (c) Just see your doctor when you can  (d) You don’t need to ring an ambulance  (e) I don't know  **20.Rehabilitation can assist someone who has suffered**  (a) Loss of movement  (b) Loss of speech or language  (c) Loss of balance  (d) All of the above  (e) I don't know | |
| **Soto-Cámara et al., 2020** | | | 1. “What stroke warning signs are you aware of?”   Valid warning signs of a stroke were as follows:   1. loss of strength or sudden weakness in the face, arm, or leg, especially on one side of the body. 2. A sensitivity disorder in the face, arm, or leg. 3. Sudden confusion or difficulty in speaking or understanding language 4. A sudden change in one’s sight in one or both the eyes 5. A sudden problems in walking; dizziness and/or loss of balance or coordination 6. A sudden, intense headache without a known cause. | 1. “What stroke RFs are you aware of?”   The stroke RFs considered to be possible correct answers were:  old age, a previous stroke, high blood pressure (HBP), diabetes mellitus (DM), dyslipidemia, overweight or abdominal obesity, cardiovascular disease, active smoking habit, excessive alcohol intake, diet risk score, a sedentary lifestyle, and psychosocial factors   1. “If you think someone is having the symptoms of a stroke, which will be your first reaction?”   1- No specific action is required  2- Treatment should be sought, although not necessarily immediately  3- This is an emergency and medical attention should be sought immediately | |
| **Alhusayni et al., 2020** | | | Compared to the general population, patients with previous strokes have a______ risk of stroke recurrence   1. Lower 2. Similar 3. *Higher (Considered correct)   Compared to previous stroke, the outcome of the second stroke will be ______   1. *Worse (Considered correct) 2. Same 3. Better | †Strategies that help prevent a secondary stroke   1. Risk factors treatment (Diabetes, Hypertension, etc.) 2. Healthy diet that is low in fat and salt 3. Regular exercise 4. Usage of antiplatelet therapy and anticoagulants 5. Quit smoking 6. Weight control 7. Reduction and avoidance of alcohol | |
| **Alexandrov et al., 2019** | | | Which of the Following are Signs/Symptoms of Stroke?   1. Facial Droop 2. Arm and/or Leg Weakness 3. Shortness of Breath 4. Chest Pain 5. Irregular Heart Beat 6. Speech Difficulty 7. Numbness of Face, Arm, and/or Leg 8. Vision Changes/Blindness 9. Vomiting or Diarrhea 10. Dizziness 11. Headache | | What is the BEST FIRST ACTION that should be taken if you recognize stroke symptoms?   1. Activate the emergency (911) system 2. Go or take the patient straight to the hospital 3. Wait and see if symptoms improve 4. Call the doctor’s office |
| **Faiz et al., 2019** | | | 1. State the 3 FAST symptoms face, arm/ leg and speech, and 2 additional, less common symptoms (dizziness and headache). 2. State the common risk factors smoking, diabetes, high cholesterol, older age, unhealthy diet, and alcohol | | 1. Actions to take in the case of an acute stroke:  - call the emergency medical services - go directly to hospital  1. Changes in lifestyle behaviour.  - Yes - No/unsure |
| **Faiz et al., 2018** | | | 1- Do you believe that stroke is a serious disorder?   - Yes - No   2- Do you believe that time is of importance for stroke treatment?   - Yes - No | | 3- List as many stroke risk factors as you can  4- List as many stroke symptoms  5- List as many stroke treatment options |
| **Wang et al., 2018** | | | **Common sense**   - Brain is affected in stroke. - Stroke is caused by blockage or rupture of cerebral blood vessels. - You need immediate medical attention after onset. - Stroke is a serious disease leading to death or disability.   **Sudden warning signs**   - Weakness of one of four limbs - Face asymmetry - Numbness (any part of your body) - Vision disturbances in 1 or both eye(s) - Difficulty in speaking, understanding, or slurred speech - Dizziness - Loss of balance - Consciousness disturbances - Headache | | **Risk factors that increase the possibility of stroke**   - Hypertension - Diabetes mellitus - Hyperlipidemia - Smoking - Obesity - Lack of exercise - Unhealthy lifestyle - Previous stroke attacks   **Post-treatment dos-and-don'ts**   - You need long-term medication as directed. - You should be put on active rehabilitation therapy after onset. - You should constantly monitor and control your blood pressure, blood lipid and blood sugar. |
| **Mellon et al., 2016** | | **SAQ** | Name as much stroke symptoms as you can | | Name as much stroke risk factors as you can |
|  |  | **RSQ** | 1. When you first experienced your symptoms how serious did you think they were?  2. How anxious (distressed or upset) were you by your symptoms when you first noticed them?  3. How much ability to control your symptoms did you think you have?  4. Delayed because your were embarrassed to get help | | 5. Delayed because your feared what might happen  6. Delayed because you did not recognize your symptoms as stroke symptoms  7. Delayed because you did not want to trouble anyone  8. Delayed because you did not know the symptoms of a stroke  9. Delayed because you did not realize the importance of your symptoms |
| **HA et al., 2016 (STAT)** | | | “If this happened to you or an adult friend/relative, what would you do?”  (1) call 911 immediately  (2) call doctor’s office immediately  (3) wait 1 hour and then decide  (4) wait 1 day and then decide   1. Vision suddenly blurred in one eye. 2. Her limbs suddenly become poor coordinated, e.g. unable to take keys out of the pocket. 3. When we are watching TV, she says her eyes seeing double vision. She feels dizzy and nauseous, and suddenly grabs my arm tightly. But she has no drunkard look. 4. Sudden numbness in one leg. 5. Sudden dizziness. 6. People speak alien language for few minutes that I can’t understand, and they don’t understand mine either. 7. I realize that my voice like a drunk while answering the phone. No improvement after at-tempting to speak. Actually I have no alcohol at all. 8. When sitting and watching TV, my left leg starts feeling numbness. The sensation of leg is strange, like deep sleep. I try rubbing and shaking the leg, but signs can’t be got rid of. | | 1. Sudden arm weakness, single-sided in particular. 2. I see he is trying to take his lunch by himself, but food keep dropping out from right month side. He never has this condition before. 3. Sudden cloudiness of consciousness. 4. Sudden dizziness with blurred vision 5. Sudden difficulties understanding. 6. Suddenly I can’t reach wallet due to immobility of the right arm. Mouth side starts dribbling, and I try my best to call my husband for help, but I can’t express. 7. Sudden unknown severe headache 8. Sudden speech difficulties 9. I notice that he keeps using his hand to cover the eye and blinking. He tells me that “I can't see”. It gets normal after a few minutes. 10. Facial muscles feel weak suddenly, particularly on one side. 11. Arms and facial muscles feel weak suddenly, particularly on one side, with speech difficulties. 12. Suddenly my right arm is unable to move at all 13. A sudden loss of coordination ability. |
| **Wongwiangjunt et al., 2015** | | | 1. What was the symptom that urged you to come to the hospital?  2. What was your thought about what happened to you while you were having the symptom?  3. Have you known about stroke?  4. Were you aware of having a stroke at that moment? | | 5. What did you do first after the symptom occurred?  6. How did you get to the hospital?  7. How long did you wait and observe the symptom before you decided to come to the hospital? |
| **Sanders et al., 2014** | **SPER** | | 1. Do you know the warning signs for stroke? 2. What are your personal risk factors for stroke that can be modified to lower your risk of another stroke? 3. What will you do if you develop symptoms of a stroke? | | 1. Do you know the medication you were prescribed to prevent another stroke? 2. What type of stroke did you have? |
|  | **S-TOFHLA** | | NS | |  |
| **Sundseth et al., 2014** | | | 1. ‘‘Which stroke symptoms do you know?’’ | | 1. ‘‘Which stroke risk factors do you know?’’ |
| **Mosley et al., 2013** | | | Participants were asked three questions regarding stroke symptoms, their assessment of the problem, and action taken prior to the ambulance call | | |
| **Zeng et al., 2012** | | | **Warning signs**   - Sudden numbness or weakness - Sudden confusion or trouble speaking - Sudden vision trouble - Sudden dizziness or trouble walking - Severe unexplained headache   **Stroke/transient ischaemic attacks (TIA) risk factors**   - Hypertension - Hyperlipidaemia - Smoking - Lack of regular exercise - Unhealthy diet | | - Overweight - Ischaemic heart disease - Excessive alcohol consumption - Family history of cardiovascular disease - Carotid stenosis - Suffering previous stroke/TIA - Atrial fibrillation - Diabetes - Metabolic syndrome - Chronic pressure   **Response**   - Call emergency service |
| **Sloma et al., 2010** | | | **Which one of these factors might increase or reduce the risk of stroke?**  Hypertension  Hyperlipidaemia  Smoking  Regular exercise  Overweight  Ischaemic heart disease  Older age  Excessive alcohol consumption | | Family history of cardiovascular disease  Carotid stenosis  Suffering previous stroke/TIA  Atrial fibrillation  Diabetes  Osteoporosis  Allergy  Rheumatoid arthritis  Thyroid disease |
| **Stead et al., 2008** | | | - Did you think you were having a stroke? Yes/No - Did you think time was of essence? Yes/No - Have you heard of any treatment for stroke? Yes/No - Have you heard of tPA (thrombolytics, clot busting drugs)? Yes/No | | - If yes, up to how many hours after a stroke do you think the drug can be given? --- - Do you think a stroke comes on suddenly or gradually?   A) Suddenly B) Gradually C) Either |
| **Das et al., 2007** | | | **What stroke risk factors you’re aware of:**  Smoking  Stress  Bad diet  High blood pressure  Obesity  Genetics  Lack of exercise  Alcohol  Unhealthy lifestyle  High cholesterol  Do not know | | **What stroke symptoms you’re aware of:**  Unilateral weakness/ heaviness/ numbness  Sudden confusion/ difficulty speaking/ understanding speech  Sudden difficulty in seeing in one or both eyes  Sudden difficulty walking/ dizziness/loss of balance  Headache with no known cause |
| **Lowe et al., 2006** | | | 1. Which of the following risk factors do you believe increases a person’s risk of having a stroke?   (a) Smoking  (b) Low blood pressure  (c) High cholesterol  (d) Diabetes  (e) Alcohol excess | | 2. Which part of the body does a stroke affect?  3. A stroke is a medical emergency in the same way as a heart attack.  4. Following a stroke, you are at an increased risk of having further strokes.  5. Controlling blood pressure is very important in preventing further strokes.  6. A blood pressure of 150/100 is the target blood pressure following a stroke.  7. Following a stroke, most patients should be taking aspirin every day.  8. Most tablets started in hospital can be discontinued once the patients get home. |
| **Croquelois & Bogousslavsky, 2006** | | | 1. (‘‘Do you think that hypertension, cigarette smoking, diabetes and/or hypercholesterolaemia could have been involved in your stroke?’’) | | 1. (‘‘Have you been informed that hypertension, cigarette smoking, diabetes and/or hypercholesterolaemia are involved in stroke?’’) |
| **Smith et al., 2004** | | | 1. A stroke is caused by damage to the brain. 2. A stroke is the same as a heart attack. 3. Effects of a stroke depend on which part of the brain is affected. 4. Brain cells which are affected by a stroke may not work again. 5. Most recovery occurs in the first few weeks. 6. Physical, mental, or sexual activity will increase the likelihood of a further stroke. 7. Squeezing a ball can get your hand working again. 8. Therapy can help you move your arm and leg again. 9. A speech therapist teaches you to get in and out of bed. | | 1. Feelings of frustration are a normal reaction after stroke. 2. Depression is common following stroke. 3. A stroke may affect personality and mood. 4. Flying should be avoided after a stroke. 5. Welfare benefits are provided to everybody who has a stroke. 6. It is possible to have help with both domestic and personal care. 7. Home adaptations are provided free of charge to everyone who has a stroke. 8. A social worker can give advice on welfare benefits. |
